# Inhomogeneous distribution of *Chlamydomonas* in a cylindrical container with a bubble plume

**DOI:** 10.1242/bio.015669

**Published:** 2016-01-19

**Authors:** Yuki Nonaka, Kenji Kikuchi, Keiko Numayama-Tsuruta, Azusa Kage, Hironori Ueno, Takuji Ishikawa

**Affiliations:** 1 Graduate School of Biomedical Engineering, Tohoku University, 6-6-01 Aoba, Aramaki, Aoba-ku, Sendai 980-8579, Japan; 2 Dept. Bioengineering and Robotics, Graduate School of Engineering, Tohoku University, 6-6-01 Aoba, Aramaki, Aoba-ku, Sendai 980-8579, Japan; 3 Faculty of Education, Aichi University of Education, 1 Hirosawa, Igaya-cho, Kariya, Aichi 448-8542, Japan

**Keywords:** Aeration, Biofluid mechanics, Flow visualization, Microorganism, Swimming, Vortex

## Abstract

Swimming microalgae show various taxes, such as phototaxis and gravitaxis, which sometimes result in the formation of a cell-rich layer or a patch in a suspension. Despite intensive studies on the effects of shear flow and turbulence on the inhomogeneous distribution of microalgae, the effect of a bubble plume has remained unclear. In this study, we used *Chlamydomonas* as model microalgae, and investigated the spatial distribution of cells in a cylindrical container with a bubble plume. The results illustrate that cells become inhomogeneously distributed in the suspension due to their motility and photo-responses. A vortical ring distribution was observed below the free surface when the bubble flow rate was sufficiently small. We performed a scaling analysis on the length scale of the vortical ring, which captured the main features of the experimental results. These findings are important in understanding transport phenomena in a microalgae suspension with a bubble plume.

## Abstract (precis)

**Summary:** A substantially inhomogeneous distribution of micro algae was developed in suspension with a bubble plume. A vortical ring and vertical layers of cells were observed when the cells displayed phototaxis and motility.

## INTRODUCTION

Microalgae photosynthesize; they contribute to the ecosystem of a pond, a lake, or the ocean as a primary producer that synthesizes organic substances from inorganic ones using the energy of light, simultaneously generating oxygen, through the chemical reactions of photosynthesis. Many microalgae have motility: in particular, many eukaryotic microalgae have flagella protruding from their cell body, and are capable of swimming by beating their flagella. Microalgae are used in photobioreactors to produce biodiesel, bioethanol, and animal feed, as well as to reduce pollutants, such as NO_x_ and CO_2_ (Georgianna and Mayfield, 2012; Haruna et al., 2010; Muñoza and Guieysse, 2006). In most cases, the spatial distribution of motile microalgae in a suspension is not uniform. For example, ‘red tides’ in the ocean are caused by accumulation of microalgae, resulting in large-scale coloration of the surface water (Wyatt and Horwood, 1973). In such an accumulated region, cells intensively uptake nutrients and disturb the transmission of light. Thus, algal growth rates would be expected to be strongly affected by their own spatial distribution.

An inhomogeneous distribution of motile microalgae can be caused by behavioral responses to various stimuli, such as taxes and kinesis. Phototaxis is the movement towards or away from light stimuli. Some flagellated microalgae, such as *Chlamydomonas*, *Volvox* and *Euglena*, sense light by photoreceptor molecules in their eyespot (Witman, 1993; Hegemann, 2008), and alter their flagellar beat to turn towards or away from light (Jékely et al., 2008; Drescher et al., 2010; Goldstein, 2015). They eventually accumulate in a region with an appropriate light intensity, where they can perform photosynthesis but avoid cellular stress caused by the too strong light (Wakabayashi et al., 2011). Gravitaxis is the movement towards or away from the gravitational direction. Many swimming microalgae show negative gravitaxis: i.e. they swim upwards, on average, even with no light stimulus (Wager, 1911; Häder et al., 2005). The mechanism of gravitaxis can be explained by the bottom-heaviness of cells (Kessler, 1985a,b), the fore-aft body asymmetry of cells (Roberts, 1970, 2006), or physiological sensing of gravitational stimuli by mechanosensitive ion channels (Lebert et al., 1997). When cells accumulate in a suspension due to taxis, bioconvection can be generated. The basic mechanism of bioconvection is analogous to that of Rayleigh–Benard convection, in which an overturning instability develops when the upper regions of fluid become denser than the lower regions (Pedley and Kessler, 1992; Hill and Pedley, 2005; Williams and Bees, 2011).

Cell responses to various flow fields have been investigated widely (Lauga and Powers, 2009; Stocker, 2012; Guasto et al*.*, 2012). Kessler (1985a,b) showed experimentally that cells swim towards regions of locally downwelling fluid and away from upwelling fluid. The migration mechanism was explained by the balance of two torques: the gravitational torque due to the bottom-heaviness of cells, and the viscous torque generated by the background vorticity. Durham et al. (2009) investigated the effect of shear flow on the motion of microalgae. They showed that an accumulated layer of cells was formed when the vertical migration of cells was disrupted by hydrodynamic shear. They concluded that the mechanism of cell trapping was responsible for the thin layers of phytoplankton commonly observed in the ocean. The effect of vortex arrays on cell distribution was investigated by Durham et al. (2011). They showed that cell motility within a steady vortical flow resulted in tightly clustered aggregations of cells. Moreover, Durham et al. (2013) and De Lillo et al. (2014) investigated the effects of turbulence on cell distribution. They concluded that hydrodynamics torques due the turbulence caused microscale patches of motile phytoplankton. These results clearly illustrate the importance of fluid mechanics in the distribution of motile algal cells.

Although the effects of shear and turbulence on the inhomogeneous distribution of microalgae have been reported, those of a bubble plume have remained unclear. A bubble plume is often used in culturing microalgae to enhance mass transport in a suspension and keep a homogeneous growth rate by circulating the cells in a container, and providing nutrients such as CO_2_ and O_2_. The length scale of the bubble plume diameter is broad: in the order of millimeters under laboratory culturing conditions, centimeters in a photobioreactor, and meters in a pond. In this study, we focused on millimeter-scale bubble plumes so that the distribution of cells in a container could be visualized clearly. We used *Chlamydomonas* as model microalgae, and investigated the spatial distribution of cells in a cylindrical container with a bubble plume. We also performed a scaling analysis to explain the vortical distribution of cells found in the experiments.

## RESULTS

### Effects of cell motility and photo responses

**Fig. 1.**
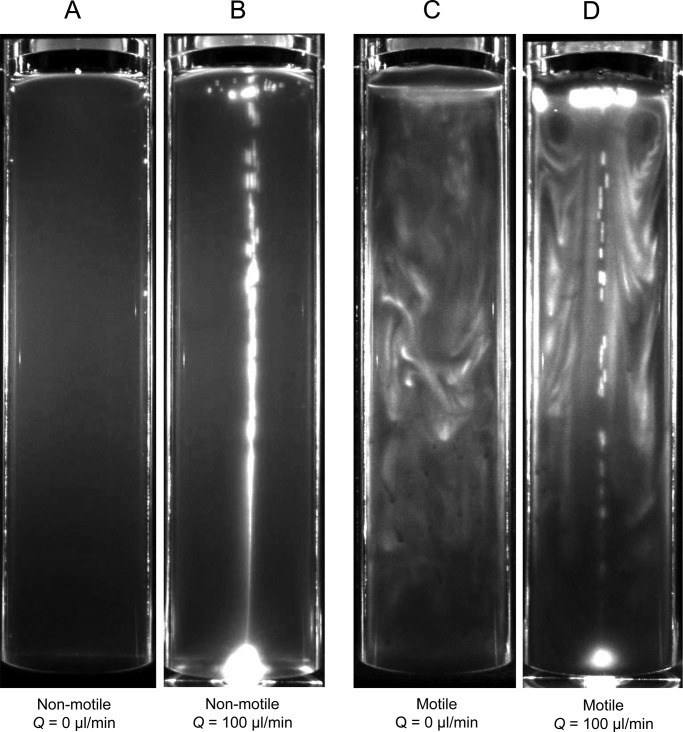
**Motility is required for inhomogeneous cell distribution in a bubble plume.** Sample snapshots of non-motile (*pf14*; A,B) and motile (wt; C,D) cell distribution with bubble flow rates of *Q*=0 µl/min (A,C) or *Q*=100 µl/min (B,D), after the suspension reached the equilibrium state (cell concentration *C*=1×10^6^ cells/ml, diameter *D*=40 mm, with blue light), showing that inhomogeneous distributions were only created by motile cells. Bubbles and cells are seen in white. The aspect ratio of the images was not modified throughout the paper.

We first compared the distribution of cells with and without motility. As described in Materials and Methods, we used the wild-type cells (wt) of *Chlamydomonas* *reinhardtii* as motile cells. As non-motile cells, we used *pf14* mutants lacking radial spokes and spoke-associated components in the flagellar axoneme, which results in a severe defect in motility (Diener et al., 1993). Fig. 1A shows a sample snapshot of the non-motile (*pf14*) cell distribution without a bubble plume, i.e. *Q*=0, when the blue light for phototaxis was illuminated at the top (*C=*1×10^6^ cells/ml, *D*=40 mm). We see that the cells were distributed almost homogeneously in the container. Because the cells had no motility, they could not swim towards the blue light. Fig. 1B shows the non-motile cell distribution with *Q*=100 µl/min, in which the bubble plume is seen in white at the cylinder center. Even with the bubble plume, we again see an almost homogeneous distribution of cells. Thus, without cell motility, no obvious pattern could be generated with the settings used, and the cells behaved like passive tracers.

In the case of motile cells (wild-type), the distribution changed dramatically, as shown in Fig. 1C and D where the cells are seen as white. Motile cells tended to swim towards the blue light due to phototaxis, in contrast with the non-motile cells. When *Q*=0 (Fig. 1C), some accumulated patches of cells formed in the suspension, and bioconvection was generated. The bioconvection patterns appeared chaotic in time and space throughout the container. When *Q*=100 µl/min (Fig. 1B), the chaotic bioconvection patterns around the cylinder center, where the bubble plume was located, were destroyed. Near the top free surface, a pair of vortical distributions of cells was clearly evident. The vortical distribution was almost axisymmetric, constructing a vortical ring of cells. Such a ring pattern has not been reported in previous studies, to our knowledge. These results illustrate that an inhomogeneous distribution of cells can develop in the case of motile cells, and the cell distribution is strongly affected by the bubble plume.

**Fig. 2.**
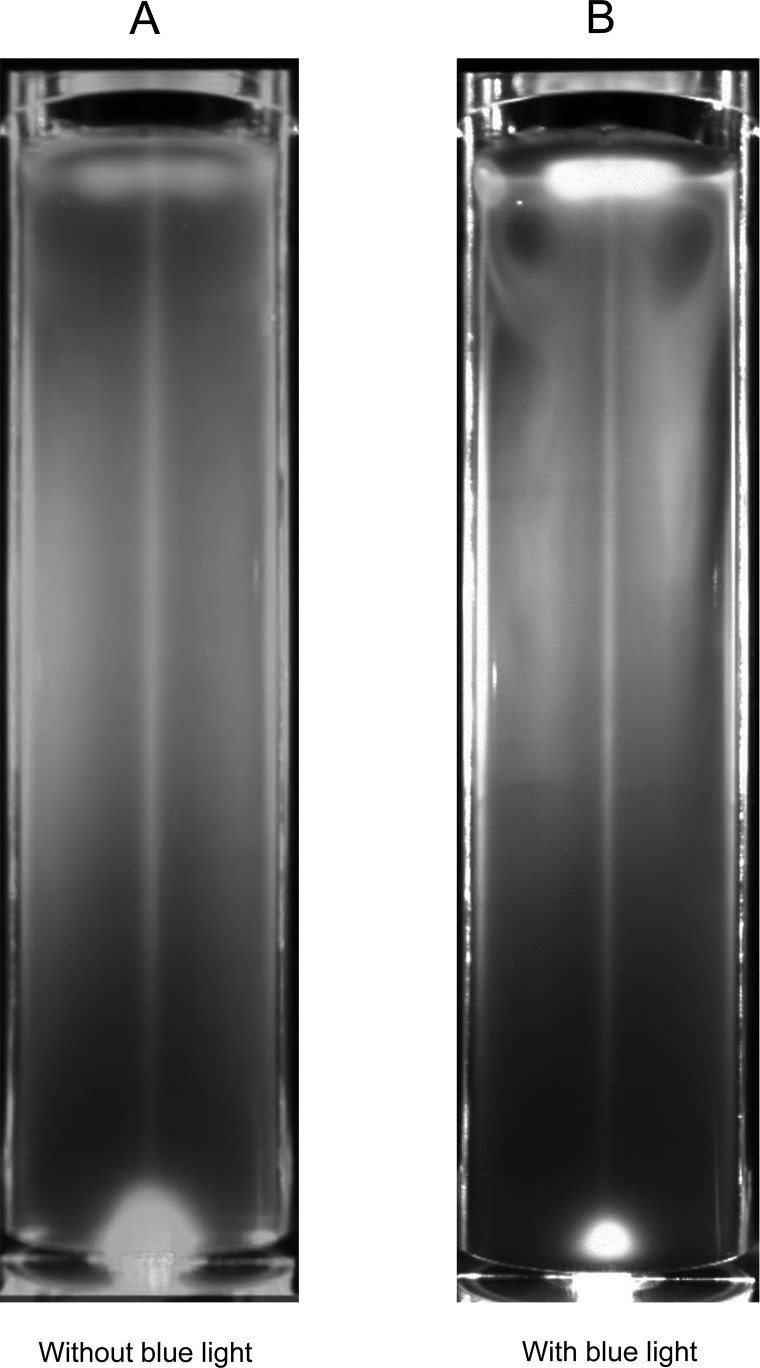
**Phototaxis is required for vortical distribution.** Time-averaged image of motile cells (wt) with (A) or without (B) blue light for phototaxis (cell concentration *C*=1×10^6^ cells/ml, bubble flow rate *Q*=100 µl/min, diameter *D*=40 mm). Inhomogeneous distributions were created with or without phototaxis, although this was stronger with phototaxis. However, vortical distribution was only observed with phototaxis. The images are averaged over 10 min for three independent experiments.

Next, we compared the distribution of cells with or without phototaxis. Fig. 2 shows time-averaged images of motile cells with or without the blue light for phototaxis (*Q*=100 µl/min, *C*=1×10^6^ cells/ml, *D*=40 mm). The images were averaged over 10 min for three independent experiments. We see that motile cells without phototaxis (Fig. 2A) generated inhomogeneous distributions on time average, although the inhomogeneity was not as strong as that with phototaxis (Fig. 2B). The vortical distribution was observed only in the case with phototaxis. These results illustrate that both motility and photo responses play major roles in the development of inhomogeneous distributions of cells.

### Effect of bubble flow rate and cell density

**Fig. 3.**
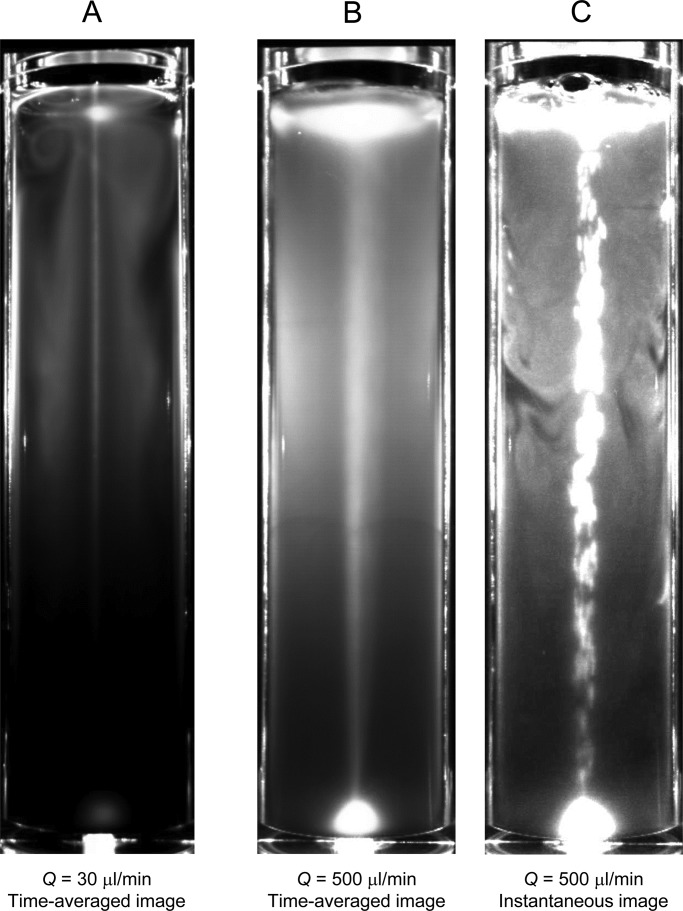
**Different bubble flow rates affect vertical distribution of motile cells.** Effect of flow rate on the distribution of motile cells (wt), where *Q* is the bubble flow rate (cell concentration *C*=1×10^6^ cells/ml, diameter *D=*40 mm, with blue light).

The effects of bubble flow rate on the distribution of cells are shown in Fig. 3 (motile cells, *C*=1×10^6^ cells/ml, *D*=40 mm, with blue light). When *Q*=30 µl/min (Fig. 3A), a vortical distribution, similar to Fig. 2B, was observed near the free surface steadily. In the middle of the container, the cells were distributed vertically between the bubble plume and the cylinder wall. A similar vertical distribution is also seen in Fig. 2B. We discuss the mechanism of vertical layer formation in the next section, ‘Vertical layers of cells in the middle of the container’.

When *Q*=500 µl/min, a vortical distribution of cells disappeared in the time-averaged image (Fig. 3B). This is because the flow generated by the bubble plume was strong enough to mix the entire contents of the container. In the instantaneous image (Fig. 3C), however, some inhomogeneity is observed, especially near the cylinder wall. We can think of several possible mechanisms to explain this phenomenon; sedimentation, phototaxis, gravitaxis, inertia induced migration and size induced migration. By considering the magnitude of the generated velocity, we suspect that the size induced migration might play an important role. The cells had a radius of ∼5 µm, and were unable to follow streamlines closer than 5 µm to the wall. Thus, the near-wall region with inward radial velocity from the wall may tend to have fewer cells than other regions. These results indicate that inhomogeneity of cells does not disappear completely even under high-bubble-flow-rate conditions, especially near the wall.

**Fig. 4.**
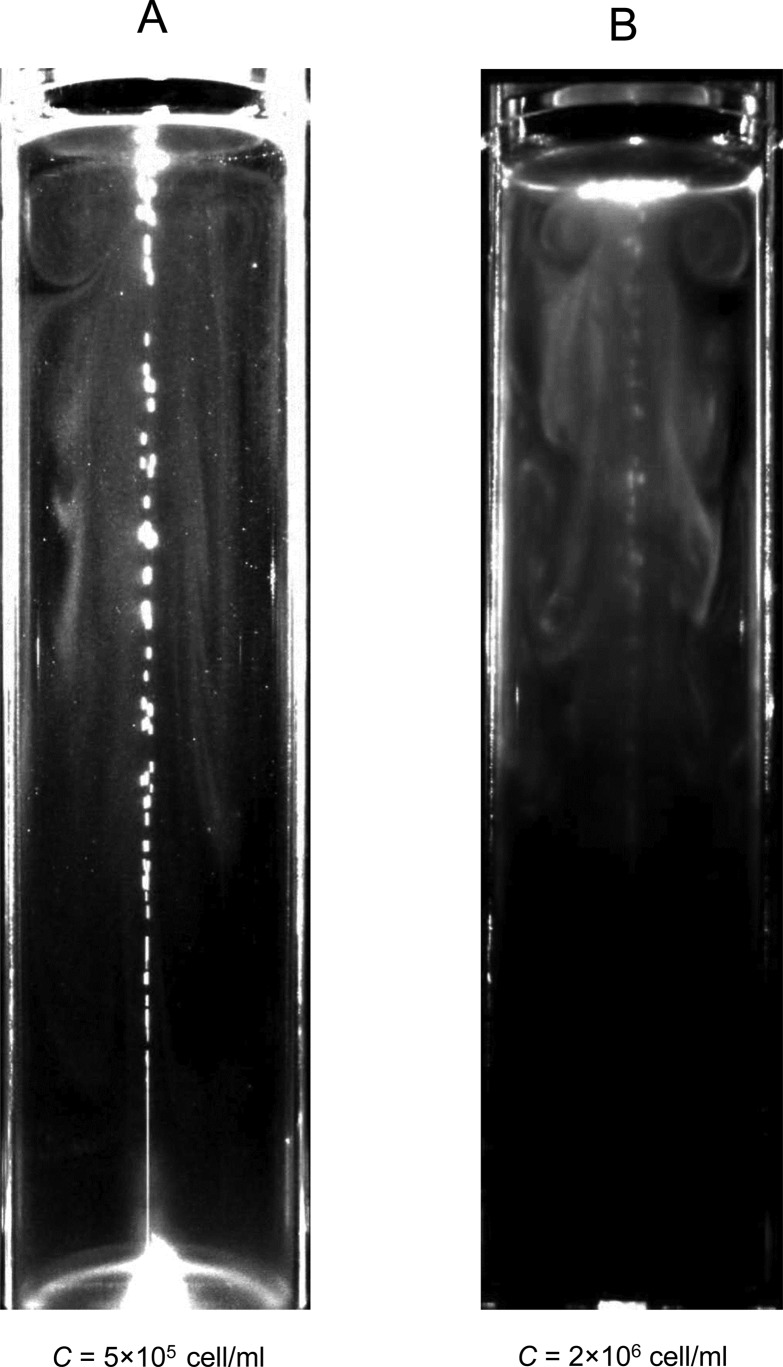
**Increased cell suspension density leads to a smaller vortical ring distribution.** Instantaneous images showing that in a lower density of cells (*C*=5×10^5^ cells/ml), vortical distribution near the free surface extends to the cylinder wall. However, in a higher density of cells (*C*=2×10^6^ cells/ml), the vortical distribution did not reach the wall, indicating a smaller vortical ring (bubble flow rate *Q*=100 µl/min, diameter *D*=40 mm, with blue light).

We also investigated the effect of the number density of cells, as shown in Fig. 4 (*Q*=100 µl/min, *D*=40 mm, with blue light). When *C*=5×10^5^ cells/ml, the vortical distribution near the free surface extended to the cylinder wall. When *C*=2×10^6^ cells/ml, however, the vortical distribution did not reach the wall, indicating a smaller vortical ring. Basically, we observed a smaller vortical ring of cells with denser suspensions, which will be discussed in the later section, ‘A vortical ring of cells near the top free surface’.

### Vertical layers of cells in the middle of the container

**Fig. 5.**
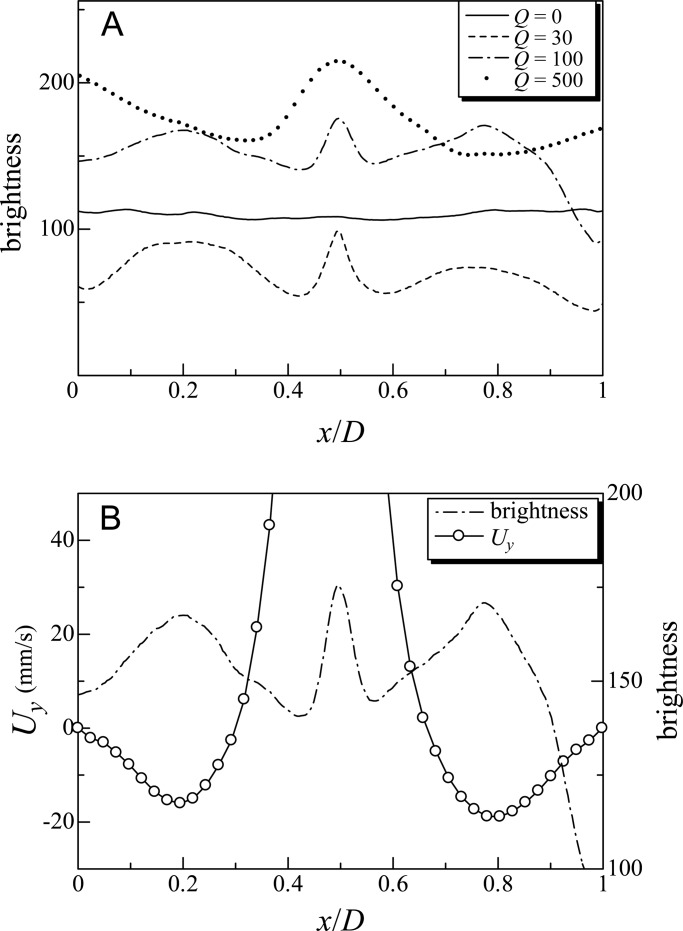
**Investigation into the mechanisms of vertical layer formation.** (A) Effect of bubble flow rate *Q* on the brightness, and (B) changes in brightness and vertical velocity (*U_y_*) when *Q*=100 μl/min, across a range of vertical velocity of background flow (*x/D*; where *x* indicates the horizontal direction of motile cells, cell concentration *C*=1×10^6^ cells/ml, diameter *D*=40 mm, with blue light). Brightness of time-averaged images is shown, indicating the cell distribution. The data is averaged in the depth direction from 35 to 70 mm from the top.

In Fig. 2B and Fig. 3A, vertical layers of cells are observed in the middle of the container. In this section, we quantify the cell distribution, and discuss the mechanism of the layer formation. Fig. 5A shows the distribution of brightness in the horizontal direction, taken by *x*, in the time-averaged images of cell distribution (*C*=1×10^6^ cells/ml, *D*=40 mm, with blue light). *x*/*D*=0.5 indicates the center of the cylinder, while *x*/*D*=0 and 1 indicate the cylinder wall. The brightness was averaged in the height direction, taken by *y*, from 35 to 70 mm from the top. A large brightness value indicates a high density of cells or a bubble plum at around *x*/*D*=0.5. The brightness was almost constant when *Q*=0, indicating that the vertical layer could not be formed without the bubble plume in the present settings. When *Q*=30 and 100 µl/min, the cells clearly accumulated around *x*/*D*=0.2 and 0.8: i.e. between the bubble plume and the cylinder wall. When *Q*=500 µl/min, however, cells tended to accumulate near the wall.

To elucidate the mechanism of vertical layer formation, we measured the background velocity field. Fig. 5B shows the vertical velocity component *U_y_* averaged in the height direction, from 35 to 70 mm from the top. *U_y_* was measured in pure water without cells under the condition of *Q*=100 µl/min. The results show that strong upward flow was induced around *x*/*D*=0.5 due to the bubble plume, whereas downward flow was induced around *x/D*=0.2 and 0.8. Fig. 5B clearly illustrates that the region of downwelling fluid was identical to the region of high cell density. Cells around the downward flow experienced hydrodynamic torque, due to the background vorticity, making them orient towards the center of the downward flow. Cells also experienced counter torque due to bottom-heaviness and fore-aft body asymmetry. The balance of the two torques enhanced the focusing of cells towards the downwelling fluid. The basic mechanism is the same as in the experiments of Kessler (1985a,b), i.e. gyrotaxis, though we had both downwelling and upwelling fluids in a single cylindrical container.

### A vortical ring of cells near the top free surface

**Fig. 6.**
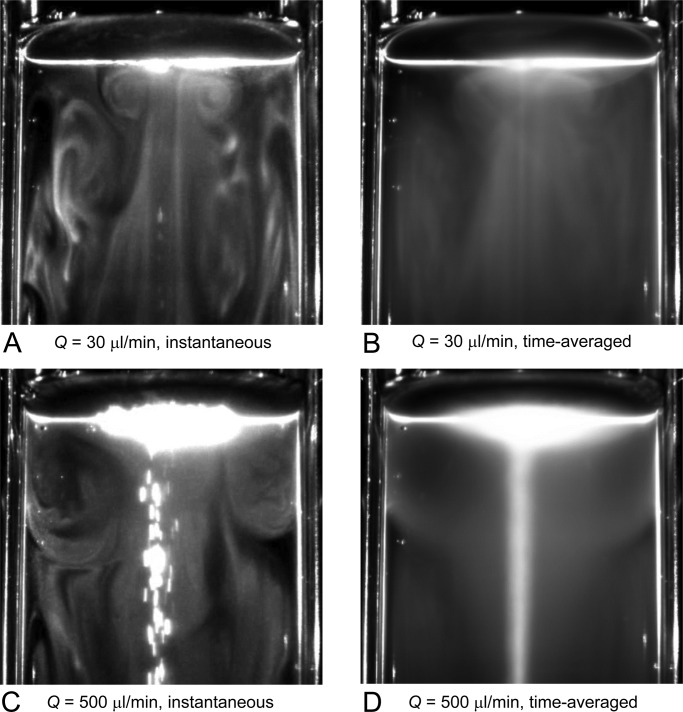
**Length scale of the vortical ring of cells increases with bubble flow rate.** Distribution of motile cells (wt) in a container with diameter *D*=80 mm (cell concentration *C*=1×10^6^ cells/ml, with blue light). Each figure shows instantaneous (A,C) or time-averaged (B,D) image of upper half of the container with bubble flow rate *Q*=30 µl/min (A,B) or 500 µl/min (C,D).

Fig. 6 shows the vortical distribution of cells in a container with a diameter of *D*=80 mm (*C*=1×10^6^ cells/ml, with blue light). The size of the vortical ring was much smaller than *D* when *Q*=30 µl/min (Fig. 6A,B). When *Q*=500 µl/min (Fig. 6C,D), however, the vortical ring expanded to the size of *D*. These results indicate that the length scale of the vortical ring of cells increased as the bubble flow rate increased.

**Fig. 7.**
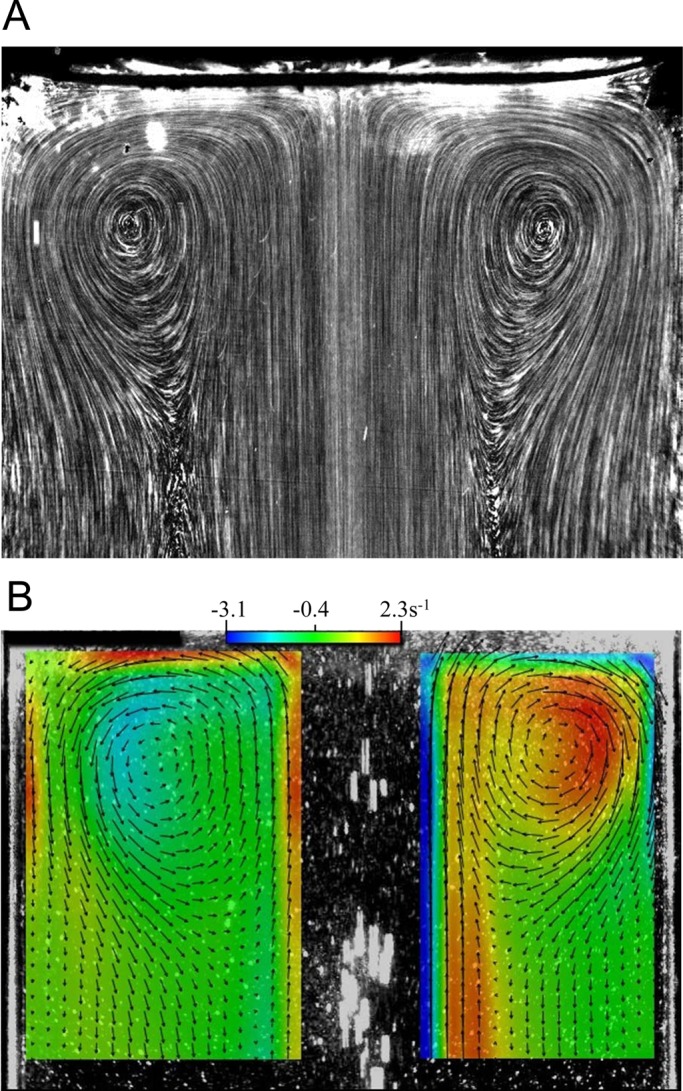
**Using tracer particles to investigative the background flow generated by bubbles.** (A) The trajectories of tracer particles used to visualize the background flow generated by bubbles with bubble flow rate *Q*=100 ml/min, in the absence of cells. Upper region of the container is shown (diameter *D*=40 mm). (B) Velocity vectors and vorticity analysis of A, color indicates the vorticity.

Was the length scale of the vortical ring of cells equivalent to that of the background vortex? To address this, we measured the center-to-center distance *L* between two vortexes. Fig. 7A shows the trajectories of tracer particles in pure water without cells. The centers of two vortexes near the top free surface are clearly evident. The images were analyzed by particle image velocimetry (PIV), and the velocity vectors and the vorticity were derived. The center of a vortex was defined as the position of maximum or minimum vorticity. The same PIV analysis was performed for suspensions of cells, and the center of the vortical distribution of cells was also defined as the position of maximum or minimum vorticity.

**Fig. 8.**
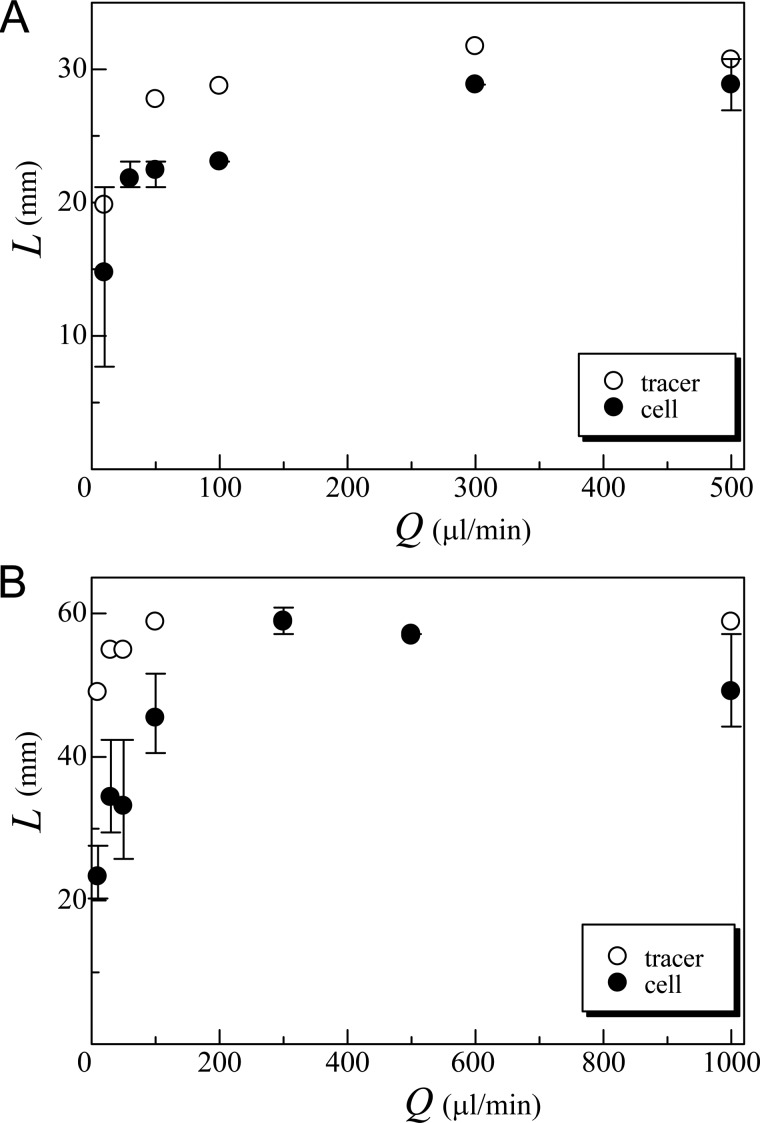
**Center-to-center distance (*L*) of the vortical ring of cells is smaller than that of the background flow, especially under low flow rate.** Two kinds of containers with diameter *D*=40 mm (A) and *D*=80 mm (B) are examined across a range of bubble flow rates *Q* (cell concentration *C*=1×10^6^ cells/ml, with blue light). The center-to-center distance *L* between the bubble flow (tracer) and the cell distribution (cell) shows that cells accumulate near the free surface then sediment relative to the surrounding fluid, which generated a downward motion earlier than in the background flow. Error bars indicate the maximum and minimum values

Fig. 8 shows a comparison of the center-to-center distance *L* between the background vortex (tracer) and the vortical cell distribution (cell). Two kinds of containers (*D*=40 and 80 mm) were examined with various bubble flow rates (*C*=1×10^6^ cells/ml, with blue light). The results show that *L* of the vortical ring of cells was smaller than that of the background flow, especially in the small *Q* regime. This is because cells accumulated near the free surface then sedimented relative to the surrounding fluid, which generated a downward motion earlier than in the background flow. The maximum value of *L* was restricted by *D*, so the value of *L* was almost constant when *Q* was sufficiently large.

## DISCUSSION

In order to understand the basic mechanics of the formation of the vortical distribution of cells, we perform a simple scaling analysis in this section. In the present experimental settings, *C. reinhardtii* showed phototaxis, gravitaxis and gyrotaxis. All taxes made cells to swim vertically upward on average and could not explain the downward motion of cells. We thus assumed that a sinking plume in the vortical ring of cells was generated mainly by the sedimentation effect, when the background flow was sufficiently weak.

The length scale of center-to-center distance *L* of the vortical ring of cells may be expressed by using a velocity scale for cells to move horizontally due to the background flow and a time scale for homogeneously distributed cells to form a falling plume. We thus derive the velocity and the time scales in the followings.

Under a constant bubble diameter condition, the bubble flow rate *Q* is proportional to the number of bubbles generated per unit time. Each bubble experiences the same buoyancy force, which has to balance with the viscous drag force once the upward bubble velocity reaches the terminal velocity. The total drag force may be proportional to the number of bubbles, which is assumed as proportional to *Q*. Since the viscous drag force is the driving force of the background flow in the container, the driving force *F_d_* can be assumed as *F*_*d*_∝*Q*.

By considering the conservation of momentum in the vertical direction, the upward driving force *F_d_* has to balance with the downward viscous friction force on the cylindrical wall *F_v_*, i.e. *F_d_*=*F_v_*, when the flow is in the steady state. *F_v_* can be expressed by a product of the shear stress *τ* and the surface area *A*, i.e. *F_v_=τ A*, and *A* is proportional to the radius of the cylindrical container *R* under the fixed depth condition. *τ* is proportional to the velocity gradient 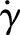, under a constant viscosity condition, and 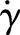 may be proportional to the background velocity scale *U* divided by *R*, i.e. 
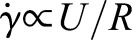
. By combining the scaling for the driving force and the friction force, we have *U*∝*Q*.

The time scale for homogeneously distributed cells to form a falling plume has been investigated by Jánosi et al. (1998) and Kitsunezaki et al. (2007). Jánosi et al. (1998) defined ‘delay time’ as the time duration from the end of mixing of the suspension until bioconvection pattern occurs. They investigated a suspension of *Bacillus subtilis* in a shallow chamber, and found that the delay time was independent of the width of the chamber and inversely proportional to the cell concentration. Kitsunezaki et al. (2007) investigated the delay time using a suspension of *Paramecium tetraurelia* in a chamber with 12 mm depth. They also found that the delay time was inversely proportional to the cell concentration, when the cell concentration was sufficiently large. In the present scaling, therefore, we assume that the delay time *T* is inversely proportional to the number density of cells, i.e. *T*∝*C*^−1^.

We further assume that the center-to-center distance *L* of the vortical ring of cells is proportional to the product of the background velocity *U* and the delay time *T*, because *L* increases as the horizontal background velocity and the time duration to form a falling plume are increased. Moreover, in the present experimental settings, the minimum value of *L* was not zero because of the finite width of the bubble plume at the center of the container. We thus define *L_min_* as the minimum value of *L*, which depends on the experimental settings, and write the correlation as
(1)
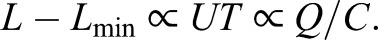

**Fig. 9.**
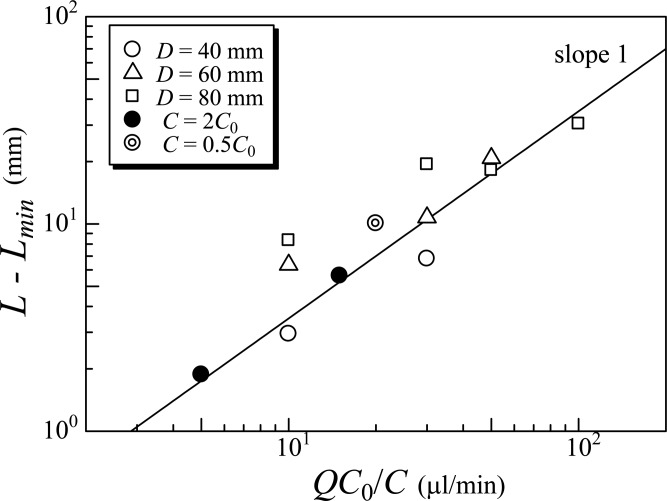
**The size of the vortical ring of cells is mainly a function of flow rate and cell density.** Center-to-center distance *L*, smaller than *D*/2, is plotted in log-log scale against *QC*_0_/*C* for all experimental conditions (diameter *D*=40, 60, and 80 mm; normalized cell concentration *C*/*C*_0_=0.5, 1, and 2, where *C*_0_=1×10^6^ cells/ml; with blue light). A fitted line of *L*−*L_min_*=0.35(*QC*_0_/*C*) with *r*^2^=0.85 and *L_min_*=15 mm (slope 1) is also drawn for comparison.

To check the above correlation, the center-to-center distance *L*, smaller than *D*/2 to avoid the wall effect, is plotted against *QC*_0_/*C* for all experimental conditions in Fig. 9 (*D*=40, 60, and 80 mm; *C*/*C*_0_=0.5, 1, and 2, where *C*_0_=1×10^6^ cells/ml). Since we did not observe *L* smaller than about 15 mm throughout the study, we set *L_min_*=15 mm. Both axes are taken in log scale, and a fitted line of *L*−*L_min_*=0.35(*QC*_0_/*C*) with *r*^2^=0.85 is also drawn in the figure for comparison. We see that the results fit well with the straight line. Fig. 9 illustrates that the size of the vortical ring of cells is mainly a function of *Q* and *C*, when the effect of the wall boundary can be neglected.

## CONCLUSIONS

In this study, we used *C. reinhardtii* as model microalgae and investigated the spatial distribution of cells in a cylindrical container with a bubble plume. The results illustrated that cells became inhomogeneously distributed in the suspension due to their motility and photo responses. Vertical layers were formed in the middle of the container, where downward flow was generated. A vortical ring of cells was observed below the free surface when the bubble flow rate was sufficiently small. The center-to-center distance *L* of the vortical ring of cells was smaller than that of the background vortex, and decreased with increasing *C* or decreasing *Q*. We performed a scaling analysis on *L*, which gave the correlation *L*−*L*_min_∝*Q*/*C*. The scaling captured the main features of the experimental results. These findings are important in understanding the inhomogeneous distribution of cells as well as transport phenomena in a microalgae suspension with a bubble plume.

## MATERIALS AND METHODS

We used *Chlamydomonas reinhardtii* (strain 137c, wild-type) as model microalgae. *C. reinhardtii* is a unicellular alga with two flagella for swimming; its body length is about 10 µm. It detects light with an eyespot and shows phototaxis. For comparison with non-motile cells, the mutant strain *pf14* of *C. reinhardtii*, which has a severe defect in flagellar motility and cannot swim, was also used. Cells were cultured at 25°C under a 12/12-h light/dark cycle in tris-acetate-phosphate (TAP) (Gorman and Levine, 1965) medium with gentle aeration. To obtain a suspension of a given cell density (0.5 to 2×10^6^ cells/ml), the number density of cells was measured with a cell counter plate (Neubauer improved type, Watson, Japan), and then diluted with fresh TAP. Experiments were carried out at the middle of the light phase and at ambient temperatures of 20°C to 27°C.

**Fig. 10.**
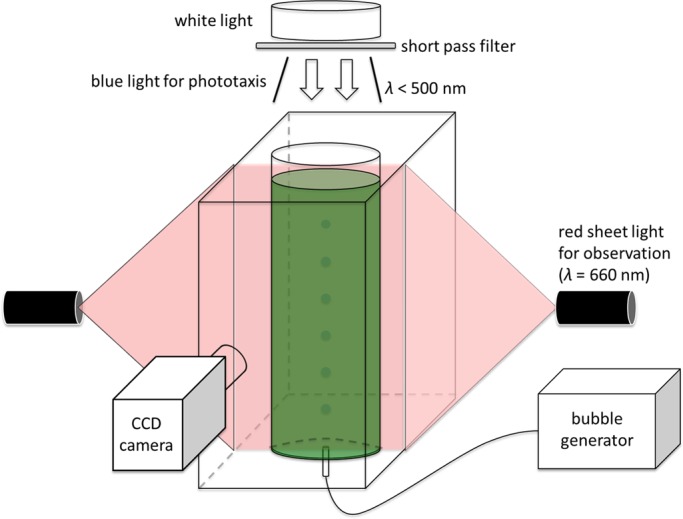
**Schematic of the experimental apparatus.** A cylindrical container, made from a rectangular acrylic block, was filled with culture fluid. The container was illuminated from both sides by two laser sheets with a wavelength of 660 nm. When we explicitly observed phototactic responses by the cells, the container was illuminated by LED light from the top. Cell distribution was recorded with a CCD camera.

Fig. 10 shows a schematic of the experimental set-up. A cylindrical container with diameter *D=*40, 60, or 80 mm was made from a rectangular acrylic block. It was filled with culture fluid with a number density of *C*=0.5, 1, or 2×10^6^ cells/ml. The depth of the culture fluid was 140 mm throughout the study. To visualize the cell distribution, the container was illuminated from both sides by two laser sheets with a wavelength of 660 nm, which are directed perpendicular to the side of the rectangular acrylic block. The wavelength of 660 nm does not induce phototactic behavior in *C. reinhardtii* (Witman, 2009), and thus we could observe their behaviors without any phototactic response. When we explicitly observed phototactic responses by the cells, the container was illuminated by LED light (LA-HDF158AS, Hayashi Watch-Works, Tokyo, Japan) from the top, as shown in Fig. 10. A low-pass filter transmitting <500 nm (SHX500, Asahi Spectra, Tokyo, Japan) was attached to the LED, so that the red light for observation and the blue light for phototaxis (Witman, 2009) could be split in recording. The light intensity of the blue light was 200 µmol/(m^2^s) at the top surface of the suspension. The illumination was slightly weakened as the depth increased, because the cells absorbed the illumination light. In order to avoid the slightly non-uniform illumination effect, we measured brightness of the image at the same depth or binarized the images for particle image velocimetry (PIV) analysis. Cell distribution was recorded with a CCD camera (FR210-M, Flovel, Tokyo, Japan) with a frame rate of 1 fps, which is directed perpendicular to the front side of the rectangular acrylic block. Bubbles were generated at the bottom center of the container with a cigarette filter (BBA61032, Japan Filter Technology, Ltd., Tokyo, Japan) connected to a syringe. The mean diameter of the bubbles was ∼0.77 mm. The bubble flow rate *Q* was regulated in the range from 0 to 1000 µl/min by controlling a linear motor attached to the syringe. The images were recorded for 10 min in each case after the distribution of cells reached the equilibrium state. Experiments were repeated three times for each condition. The images obtained were analyzed by PIV with commercial software (Flownizer 2D3C; Ditect, Tokyo, Japan). The PIV analysis was processed using interrogation windows of 16×16 or 32×32 pixels with 62.5% overlap. A cross-correlation method was used to calculate the velocity field.

We also analyzed the velocity field generated only by the bubble plume in the absence of cells (i.e. the background velocity field). In this case, tracer particles with a diameter of 12 µm and a relative density of 1.06 (SBX-12, Sekisui Plastics, Osaka, Japan) were suspended in pure water. Another CCD camera (HAS220, Ditect) was used to record images for 1 min with a frame rate of 50 fps. Other settings were the same as for the cell suspension cases.
